# Impact of Vaginal Carbon Dioxide Laser Therapy Alone Versus Its Combination With Oral Bioactive Collagen Peptides, Ultra‐Low Molecular Weight Hyaluronic Acid, and Other Functional Components on the Genitourinary Syndrome of Menopause: A Cohort Pilot Study in Italy

**DOI:** 10.1111/jocd.70474

**Published:** 2025-09-27

**Authors:** Alessandro Tafuri, Andrea Panunzio, Claudia Rita Mazzarella, Michela Tricarico, Ezio Michele Tricarico

**Affiliations:** ^1^ Department of Urology “Vito Fazzi” Hospital Lecce Italy; ^2^ Department of Obstetrics and Gynecology “Giuseppe Tatarella” Hospital Cerignola Italy; ^3^ Department of Gynecology “Amalthea” Diagnostic Center Lecce Italy

**Keywords:** bioactive collagen peptides, CO_2_ laser therapy, genitourinary syndrome of menopause, menopause, MonaLIsa, vaginal atrophy

## Abstract

**Background:**

Genitourinary syndrome of menopause (GSM) includes clinical manifestations attributed to estrogen deficiency affecting the genitourinary tract of postmenopausal women. Treatment may require a multifaceted approach, including patient education, lifestyle modifications, physical, as well as hormonal and non‐hormonal therapies.

**Aims:**

To evaluate the efficacy and tolerability of vaginal CO_2_ laser therapy (MonaLisa Touch) combined with an oral food supplement containing bioactive collagen peptides (BCP) and other functional components for GSM treatment. We hypothesized that this combination would enhance GSM symptom relief.

**Patients/Methods:**

Twenty postmenopausal women with GSM were divided into two groups. Group 1 (*n* = 10) underwent three sessions of vaginal CO_2_ laser treatment alone, while Group 2 (*n* = 10) received the same laser treatment in addition to oral food supplementation. Improvements in vaginal health (Vaginal Health Index—VHI), vaginal pain (Visual Analogue Scale—VAS), and sexual function (Female Sexual Function Index—FSFI), along with patient satisfaction and tolerability, were evaluated.

**Results:**

Both groups showed improvements in VHI, pain scores, and FSFI, with Group 2 displaying more significant gains. Compared to Group 1, Group 2 had greater median differences in VHI (Δ = 11.00 vs. Δ = 8.50, *p* = 0.005) and VAS (Δ = −7.00 vs. Δ = −5.50, *p* = 0.017). Similarly, FSFI scores increased meaningfully in both groups, more so in Group 2 (from 53.50 to 67.50, *p* = 0.005 vs. from 51.50 to 60.50, *p* = 0.014 in Group 1). Treatment satisfaction was also higher in Group 2 (*p* = 0.047).

**Conclusions:**

The addition of oral supplementation with BCP and other functional components to vaginal CO_2_ laser treatment for GSM offered significant benefits over laser treatment alone, improving vaginal health, reducing pain, and ameliorating sexual function. This combination therapy presents a promising non‐hormonal option for GSM management, warranting further investigation in larger, long‐term studies to confirm these preliminary findings.

## 1 Introduction

Genitourinary syndrome of menopause (GSM), previously known as vulvovaginal atrophy (VVA), is characterized by clinical manifestations attributed to estrogen deficiency impacting the genitourinary tract of postmenopausal women [1]. Signs and symptoms of GSM may include vaginal dryness, burning, itching, irritation, dyspareunia, vaginal discharge, urinary incontinence, dysuria, as well as a decrease in quality of life and sexual health, and can affect a woman's overall well‐being [1, 2, 3].

GSM treatment requires a multifaceted approach, involving the collaboration of patients and different specialized professionals, including gynecologists, urologists, physiotherapists, and psychologists: indeed, treatment options may include patient education, lifestyle modifications, physical treatments, non‐hormonal therapies, and, in some cases, hormonal treatments and hormonal receptor modulators [3, 4, 5]. Palacios et al. recently suggested adopting a sequential approach to treatment, following patients' needs and preferences over time, to improve adherence and persistence to therapy [6].

Treatments aimed at restoring metabolism and function of connective tissue, the most important of which is bio‐stimulation activating fibroblast anabolic functions, and particularly enhancing type III collagen, elastin, and hyaluronic acid (HA) production from their precursors, are considered to be an innovative approach according to the principles of antiaging medicine [7].

Intravaginal energy‐based methods, such as laser and radiofrequency, have been proposed as non‐pharmacological therapeutic options for managing GSM and have demonstrated promising results in alleviating symptoms, improving quality of life, and restoring sexual function without severe systemic adverse events [4]. Various laser systems have been used to treat GSM: the carbon dioxide (CO_2_) laser is an ablative fractional device, which promotes selective photothermolysis by targeting water molecules within cells, while the non‐ablative erbium‐doped yttrium aluminum garnet (Er: YAG) laser exhibits lower penetration and thermal effects compared to the CO_2_ laser [8]. Such treatments appear feasible options in GSM management, improving vaginal health by activating growth factors, leading to increased vascularity, collagen synthesis, production of the extracellular matrix, and thickening of the vaginal epithelium [9]; additionally, there is an increase in *Lactobacillus* and other premenopausal flora, indicating a restoration of the vaginal microenvironment towards a premenopausal state [10].

In the evolving landscape of menopausal health management, the quest for effective treatments that address a broad spectrum of symptoms without the risk associated with the use of hormones has become increasingly important. Within the non‐hormonal therapies, vaginal moisturizers and lubricants are usually employed, although they do not reverse the epithelium atrophy, may cause disruption of the vaginal ecosystem, and are often not well received by patients [11]. Moreover, few studies support the application of these treatments as therapeutic strategies [12]. There is also a need to expand therapeutic options with easy‐to‐administer, non‐hormonal oral products that can be combined with energy‐based methods to extend and amplify their efficacy.

Recently, a new food supplement aiming at restoring vulvo‐vaginal eutrophism has been marketed. It consists of specific bioactive collagen peptides (BCP, 2.5 g) combined with ultra‐low molecular weight HA (ULMW‐HA, 3–10 kDa, 75 mg) and other functional components such as a nucleotide blend. The present study aimed to evaluate and compare the efficacy and tolerability of vaginal CO_2_ laser treatment (MonaLisa Touch) alone and in combination with this oral food supplement for the treatment of GSM. We hypothesized that the combined protocol with the addition of the food supplement would be more effective in restoring vulvovaginal eutrophism and alleviating GSM symptoms.

## 2 Materials and Methods

### 2.1 Study Design and Population

The cohort study was conducted on 20 volunteer menopausal women aged > 50 years diagnosed with GSM. Before the study began, every patient gave informed consent for enrollment, data collection, and analysis. All patients had a cervical‐vaginal cytologic screening performed within the year and a negative urine culture; those who suffered from vulvovaginal infections, bladder infections, or presented with abnormal uterine bleeding were excluded from the study. Women were then sequentially allocated (1:1) in two treatment groups: Group 1, consisting of 10 patients, underwent three sessions of vaginal CO_2_ laser therapy (MonaLisa Touch), spaced 40 days apart; Group 2, also consisting of 10 patients, underwent the same three sessions of vaginal CO_2_ laser therapy (MonaLisa Touch), spaced 40 days apart, in combination with a food supplement containing 2.5 g of BCP, 75 mg of ULMW‐HA, and other functional components, starting from the first day of the study for a total of 120 days.

### 2.2 Outcomes Measurements and Timepoints

For all patients, the following data were prospectively collected in an electronic database: age (years), body mass index (BMI; kg/m^2^), educational level, marital status, pregnancies, menopause age, and any comorbidity, including a history of previous surgery. Women were evaluated at four key time points: V1, marking the start of the study and corresponding to the first session of vaginal CO_2_ laser treatment for both groups and the start of the oral food supplementation exclusively for Group 2; V2, and V3, scheduled at the second and third sessions of the laser treatment, respectively; and V4, corresponding to 40 days after the final laser treatment session and the end of oral supplementation.

The evaluation considered GSM signs, pain perception related to vaginal atrophy, and sexual function. GSM signs were assessed using the Vaginal Health Index (VHI) before each vaginal CO_2_ laser treatment session (V1, V2, and V3) and 40 days after the last one (V4). All VHI evaluations were performed by the same practitioner, who was not blinded to the participants' treatment group. The practitioner evaluated vaginal elasticity, secretions, pH level, epithelial integrity, and hydration levels. These assessments were based on predefined criteria outlined in a standardized table (as shown below). Each parameter was scored from 1 (worst scenario) to 5 (best scenario) according to objective benchmarks, ensuring consistency and minimizing subjectivity. Total scores for VHI ranged from 5 to 25, with a cut‐off of < 15 adopted to indicate an atrophic vagina. Pain perception related to GSM was assessed through the Visual Analogue Scale (VAS) (0–10) before each vaginal CO_2_ laser treatment session (V1, V2, and V3) and 40 days after the last one (V4). Sexual function was evaluated through the administration of the Female Sexual Function Index (FSFI) questionnaire, a 19‐item questionnaire assessing six domains: desire, arousal, lubrication, orgasm, satisfaction, and pain. Total scores range from 2 to 36, with higher scores indicating better sexual function. The FSFI was administered before the first vaginal CO2 laser treatment session (V1) and 40 days after the final session (V4).

As a secondary measure, we evaluated patient adherence in both groups using a dedicated satisfaction survey administered at the end of the study (V4), where scores ranged between 0 (totally unsatisfied, no perceived benefit) and 20 (totally satisfied). Additionally, for patients receiving oral supplementation with BCP and other functional components, the following aspects were investigated: flavor, modality of administration, gastrointestinal tolerability, and duration of the therapy, assigning scores ranging from 0 (totally unsatisfied) to 10 (totally satisfied) for each aspect. Furthermore, we documented any adverse events reported throughout the study.

### 2.3 Interventions

The vaginal CO_2_ laser system MonaLisa Touch (DEKA, Florence, Italy) is a fractional ablative intravaginal therapy that is delivered every 30–45 days for three consecutive months. Each treatment session lasts around 20 minutes. The settings of the micro ablative fractional CO_2_ laser used in this study for the treatment of the vaginal canal were the following: D‐Pulse mode, dot power 40 W; dwell time 1000 μs; and dot spacing 1000 μm. For the treatment of the vaginal introitus, the setting was the following: dot power 24 W; dwell time, 400 μs; and dot spacing 1000 μm. The procedure was performed in an outpatient setting and did not require any specific preparation or anesthesia. Participants were placed in a dorsal lithotomy position. It was recommended to avoid vaginal sexual intercourse for at least 3 days after the laser application to prevent an inflammatory reaction that might last up to 48 h. Only patients from Group 2 concurrently received a systemic treatment, with a commercially available oral supplement containing 2.5 g of BCP (Gelita AG, Eberbach, Germany), 75 mg of ULMW‐HA (3–10 kDa, TS‐Biotech Co. LTD, China), 250 mg of a mix composed of *Astragalus membranaceus* (Mongolian milkvetch) and *Centella Asiatica* (Gotu kola) powders (NuLiv Science Inc., Brea, CA, USA), and 100 mg of a nucleotide blend (5′‐AMP, adenosine‐5‐monophosphate, free acid; 5′‐CMP, cytidine 5′‐monophosphate, free acid; 5′‐UMP, uridine 5′‐monophosphate, disodium salt; 5′‐GMP, guanosine 5′‐monophosphate, disodium salt; 5′‐IMP, inosine 5′‐monophosphate, disodium salt; Prosol S.p.A., Madone, Bergamo, Italy). The treatment was administered once a day, starting from V1 for 120 days.

### 2.4 Statistical Analysis

The sample size for the present study was determined based on the exploratory nature of the study itself, which aimed to collect preliminary data on the efficacy and tolerability of the combined treatment protocol. As a pilot study, the primary focus was on feasibility and effect estimation rather than achieving statistical power, with findings intended to guide the design of larger, confirmatory trials.

Descriptive statistics included frequencies and proportions for categorical variables; medians and interquartile range (IQRs) were reported for continuously coded variables. Wilcoxon rank sum test and Fisher's exact test examined the statistical significance of differences in medians and proportions, respectively, between groups. All tests were two‐sided, with a level of significance set at *p* < 0.05.

R software environment for statistical computing and graphics (version 4.1.2, R Foundation for Statistical Computing, Vienna, Austria) was used for all analyses.

## 3 Results

### 3.1 Participant Baseline Characteristics

Participant characteristics are detailed in Table 1. Overall, the median age was 54 (IQR: 52–59) years, and the median BMI was 23.5 (IQR: 22.1–25.7) kg/m^2^. There were no significant differences in the demographic and clinical characteristics across the different treatment groups.

**TABLE 1:** Descriptive characteristics of the study population.

Characteristic	Overall	Group 1	Group 2	*p* ^b^
	*n* = 20 (100%)^a^	*n* = 10 (50%)^a^	*n* = 10 (50%)^a^	
Age (years)	54 (52, 59)	55 (48, 60)	54 (52, 57)	0.9
BMI (kg/m^2^)	23.5 (22.1, 25.7)	23.1 (21.4, 24.2)	25.2 (22.7, 25.8)	0.3
Educational level				0.9
Middle‐school diploma	2 (10.0%)	1 (10.0%)	1 (10.0%)	
High‐school diploma	6 (30.0%)	3 (30.0%)	3 (30.0%)	
University degree	12 (60.0%)	6 (60.0%)	6 (60.0%)	
Number of pregnancies	1 (1, 2)	2 (0, 2)	1 (1, 2)	0.9
Duration of menopause (years)	5 (3, 9)	7 (5, 10)	5 (3, 8)	0.4
Marital status				0.4
Unmarried	6 (30.0%)	4 (40.0%)	2 (20.0%)	
Married	12 (60.0%)	6 (60.0%)	6 (60.0%)	
Separated/divorced	10 (10.0%)	0 (0%)	2 (20.0%)	
Previous surgery	5 (25.0%)	2 (20.0%)	3 (30.0%)	0.9

*Note:* Group 1: vaginal carbon dioxide (CO_2_) laser therapy (MonaLisa Touch) alone. Group 2: vaginal CO_2_ laser therapy (MonaLisa Touch) and oral supplementation of BCP and other functional components.

Abbreviation: BMI, body mass index.

^a^ Median (IQR); *n* (%).

^b^ Wilcoxon rank sum test; Wilcoxon rank sum exact test; Fisher's exact test.

### 3.2 Outcome Measurements

Adherence to the treatment protocol was 100%. All 20 participants attended all scheduled laser sessions, participants in Group 2 completed their oral supplementation as instructed, and everyone returned for the final evaluation (V4). No adverse events were reported by any participants during the study.

#### 3.2.1 Vaginal Health Index

**FIGURE 1:**
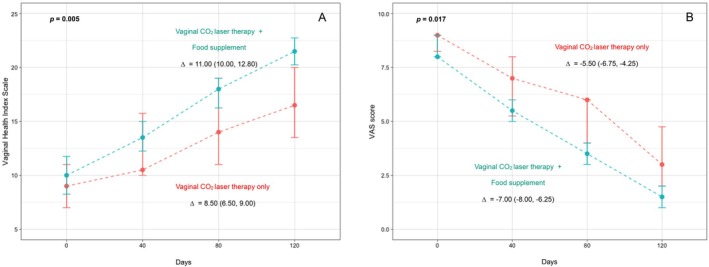
Line plots depicting (A) median (IQR) Vaginal Health Index (VHI), and (B) median (IQR) VAS score for pain in patients treated with vaginal carbon dioxide (CO_2_) laser therapy (MonaLisa Touch) alone (Group 1) or vaginal CO_2_ laser therapy (MonaLisa Touch) and oral food supplementation containing BCP and other functional components (Group 2) at V0 (baseline), V1 (40 days), V2 (80 days), and V3 (120 days).

At baseline (V1), median VHI was 9.00 for Group 1 and 10.00 for Group 2 (*p* = 0.4). Over the course of treatment, VHI for Group 1 increased to 10.50, 14.00, and 16.50 at V2, V3, and V4, respectively; improvements for Group 2 were more pronounced, with scores increasing to 13.50, 18.00, and 21.50 at V2, V3, and V4, respectively (Table 2). As shown in Figure 1A, the median difference in VHI between the final (V4) and initial (V1) assessment was greater for women receiving the combined treatment (Group 2) compared to those receiving vaginal CO_2_ laser treatment alone (Group 1): Δ = 11.00 versus Δ = 8.50, *p* = 0.005. Women from Group 2 reached a VHI > 15 (vaginal atrophy cut‐off) earlier compared to Group 1 (V3 vs. V4).

**TABLE 2:** Subjective symptoms, objective signs, and treatment satisfaction scores (median and interquartile range) at each specified time point in patients treated with vaginal carbon dioxide (CO_2_) laser therapy (MonaLisa Touch) only (Group 1) and vaginal CO_2_ laser therapy (MonaLisa Touch) and food supplement containing BCP and other functional components (Group 2).

	Group 1				Group 2			
	Vaginal carbon CO_2_ laser therapy (MonaLisa Touch) alone				Vaginal carbon CO_2_ laser therapy (MonaLisa Touch) + Food supplement			
	V1	V2	V3	V4	V1	V2	V3	V4
VHI	9.00 (7.00–11.00)	10.50 (10.00–15.75)	14.00 (11.00–19.00)	16.5 (13.5–20.0)	10.00 (8.25–11.75)	13.50 (12.25–15.00)	18.00 (16.25–19.00)	21.50 (20.20–22.80)
VAS	9.00 (8.25–9.00)	7.00 (5.25–8.00)	6.00 (4.00–6.00)	3.00 (2.00–4.75)	8.00 (8.00–9.00)	5.50 (5.00–6.00)	3.50 (3.00–4.00)	1.00 (1.00–2.00)
FSFI	51.50 (41.25–63.75)	—	—	60.50 (48.25–72.00)	53.50 (46.50–59.75)	—	—	67.50 (61.00–75.00)
Treatment satisfaction	—	—	—	11.50 (8.75–18.00)	—	—	—	18.00 (18.00–18.00)

#### 3.2.2 Pain Measurement

Baseline (V1) median VAS for pain was 9.00 in patients from Group 1 and 8.00 in those from Group 2 (*p* = 0.3). Throughout the study, these scores decreased to 7.00, 6.00, and 3.00 at V2, V3, and V4, respectively, in women from Group 1, and to 5.50, 3.50, and 1.50 at V2, V3, and V4, respectively, in women from Group 2 (Table 2). As shown in Figure 1B, the median difference in VAS for pain between the final (V4) and initial (V1) assessment was greater for women receiving the combined treatment (Group 2) compared to those receiving vaginal CO_2_ laser treatment alone (Group 1): Δ = −7.00 versus Δ = −5.50, *p* = 0.017.

#### 3.2.3 Sexual Function

Median FSFI improved from 51.50 at baseline to 60.50 at the end of the study in Group 1 (*p* = 0.014), and from 53.50 at baseline to 67.50 at the end of the study in Group 2 (*p* = 0.005), with differences between groups at final assessment not being statistically significant (*p* = 0.2; see also Table 2).

#### 3.2.4 Satisfaction and Safety Profile

Treatment satisfaction levels were higher in Group 2, with a median score of 18.00 (IQR: 18.00–18.00) compared to 11.50 (IQR: 8.75–18.00) in Group 1 (*p* = 0.047) (Table 2). Group 2 also reported high scores for each evaluated domain related to the administration of oral food supplementation, as shown in Table 3.

**TABLE 3:** Patient satisfaction scores (median and interquartile range) with treatment using the food supplement containing BCP and other functional components.

Taste satisfaction	9.00 (8.25–9.75)
Administration method	9.00 (9.00–9.75)
Gastrointestinal tolerability	10.00 (9.00–10.00)
Duration of intake	9.00 (9.00–9.75)

## 4 Discussion

Intravaginal energy‐based methods, such as the vaginal fractional CO_2_ laser, have been extensively utilized with several publications reporting favorable outcomes with minimal adverse events from their use, with persistence of positive outcomes for up to one year [13]. The procedure is generally well tolerated, with transient minor discomfort identified as the most common adverse event [14]. For example, Di Donato et al. reported that fractional CO2 laser therapy for VVA is a safe procedure with no severe complications and high patient satisfaction. Only mild discomfort was documented, mainly related to the probe introduction and rotation, and most patients were willing to repeat the procedure [15]. Gaspar et al. found that the vaginal epithelium of 92 patients treated with micro‐fractional CO_2_ laser, combined with platelet‐rich plasma, showed improvements across all three epithelial layers in pre‐and post‐treatment biopsies [16]. A similar study conducted in 2013 by Salvatore et al. observed symptomatic relief and improved VHI scores in GSM patients after 12 weeks of follow‐up [17]. A pilot study conducted on 14 postmenopausal women with GSM revealed that CO_2_ laser treatment effectively reversed epithelial atrophy and remodeled the vaginal wall's collagen, increasing type III collagen fibers, as confirmed through histological and immunohistochemical analysis [8]. The VeLVET Trial, a randomized clinical trial comparing laser therapy to vaginal estrogen therapy over six months in patients with GMS, indicated that 70% to 80% of participants were satisfied or very satisfied with either treatment without any serious adverse events registered [18]. Furthermore, a recent study confirmed the role of vaginal fractional CO_2_ laser treatment for GSM in cancer patients, asserting its value as an effective and safe therapeutic option also for gynecological cancer survivors, improving sexual life and quality of life [19]. These results were recently confirmed by a systematic review focusing on breast, ovarian, endometrial, and cervical cancer patients in whom fractional CO2 laser improves clinical symptoms and sexual function, in terms of VHI and FSFI without severe adverse events [20].

**FIGURE 2:**
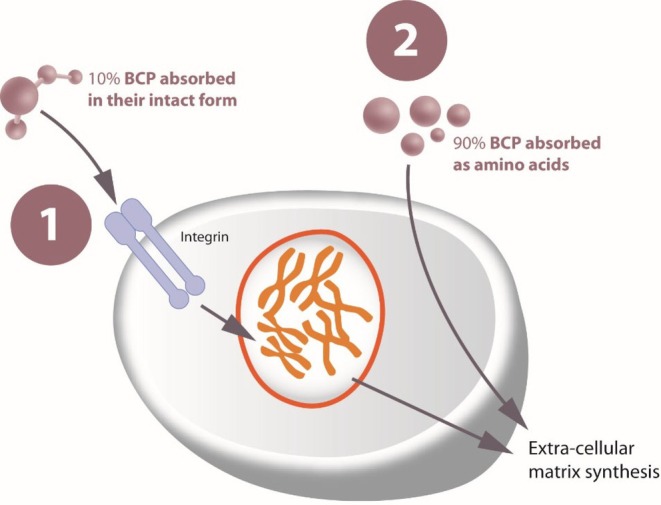
The BCPs contained in the food supplement stimulate connective tissue cells through receptor interaction. The amino acids resulting from their digestion are also important as they provide the substrate for the synthesis of new connective tissue proteins once the target cells have been directly stimulated by the BCP. This is possible thanks to the unique molecular profile of the BCP contained in the food supplement, that have an average molecular weight of 2 kDa, and greater efficacy in stimulating the synthesis of the extracellular matrix compared to other collagen peptides with similar specifications.

Among oral and non‐hormonal therapeutic options, a new oral food supplement stands out for its unique composition made of BCP (Gelita AG, Eberbach, Germany), ULMW‐HA (TS‐Biotech Co. LTD, China) and other functional components, offering a unique approach to vulvovaginal health. The specific BCP are obtained from a highly purified form of collagen protein derived from bovine skin. They are characterized by a unique stimulatory effect, which is independent of the native collagen types I, II, or III, and it is achieved through a complex multistep process that requires a specific enzymatic hydrolytic step and denaturation of the original collagen. This process results in a distinctive mass peak fingerprint, as identified by matrix‐assisted laser desorption ionization mass spectrometry (MALDI‐MS). The final BCP composition is characterized by a specific amino acid profile where the average molecular weight is 2.0 kDa. These BCP, when administered orally, have been shown to reach the bloodstream in the form of small collagen peptides and free amino acids (Figure 2) [21]. While free amino acids provide building blocks for the formation of dermal extracellular matrix proteins and for the epidermal structure, the collagen peptides act as bioactive messengers, activating different signaling pathways and stimulating human fibroblasts to synthesize collagen, proteoglycans, and elastin. In two prospective, randomized, placebo‐controlled clinical trials, Proksch et al. demonstrated that women who consumed 2.5 g/day of these specific BCP for 8 weeks experienced improvements in skin elasticity, a reduction in wrinkle volume, and an increase in the content of collagen I and elastin in their skin [21, 22]. Other clinical studies using the same specific BCP showed their efficacy in wound healing [23], nail growth and strength [24], hair thickening [25], and cellulite morphology [26]. On the other hand, hyaluronan is a glycosaminoglycan polymer typically found as a high molecular mass polymer (several thousand kDa), being a crucial component of the extracellular matrix, and playing a variety of physiological roles, including contributing to the structural integrity of connective tissues and maintaining the moisture, shape, plasticity, and firmness of the vaginal mucosa [27]. ULMW‐HA has been specifically studied for its ability to cross the intestinal epithelium, with absorption rates directly proportional to its concentration supporting the structural stability of connective tissue and providing a moisturizing effect essential for preserving the shape, flexibility, and turgidity of the vaginal mucosa [27].

Women's life expectations are increasing all over the world, and in developed countries, women expect to survive more than 30 years following natural menopause [3]. Affecting up to 50% of midlife and older women, menopause‐related genitourinary symptoms are chronic, progressive, and unlikely to improve over time, presenting a significant concern for women's health in their postmenopausal years [1]. Approximately 70% of women experiencing GSM symptoms often do not seek medical help, seeing these conditions as a natural part of menopause, and about 75% of patients with GSM question whether their healthcare providers are adequately informed about the available treatments to effectively tackle these issues and choose to self‐medicate with food supplements. Hence, it is crucial that such supplements are investigated, and the results of these studies made available to healthcare professionals, not only to better understand the treatments their patients might be using or are interested in but also to be aware of how a combined treatment approach can enhance therapeutic outcomes.

This cohort pilot study, by exploring the effectiveness of the food supplement alongside vaginal CO_2_ laser treatment, addresses a gap in current medical practice and highlights the potential of integrated treatment strategies to improve the well‐being of women with GSM. While the study's innovative approach contributes valuable insights, several limitations are acknowledged. The cohort size is relatively small, which may affect the generalizability of the findings. Furthermore, the absence of histological data to corroborate the improvements reported limits the depth of understanding regarding the biological mechanisms at play. Additionally, there is no direct comparison between the laser treatment and the novel oral supplement alone. The lack of a placebo for Group 1 and the absence of a sham laser treatment may have influenced self‐reported outcomes such as pain, sexual function, and satisfaction, as participants were aware of their treatment allocation, potentially introducing bias. Moreover, participants were not blinded to the supplementation, which could have affected perceptions and reporting. The study also did not include a comparison group treated with the supplement alone, limiting insights into the independent efficacy of the supplement. Finally, no data on the safety profile of the supplement were collected, and the study's observation period is notably short, restricting our ability to assess the long‐term efficacy and safety of the treatments. We are working on further studies to evaluate the efficacy of the food supplement alone in treating symptoms and signs of GSM in a broader population.

In conclusion, this pilot study provides insights into the enhancement of vaginal CO_2_ laser treatment (MonaLisa Touch) for GSM management through the addition of oral supplementation with BCP and other functional components, as evidenced by the objective improvements in vaginal health and sexual function, and subjective amelioration of GSM symptoms, including pain reduction.

## Author Contributions

**Claudia Rita Mazzarella:** protocol development, data collection. **Michela Tricarico:** protocol development, data collection. **Ezio Michele Tricarico:** protocol development, data collection. **Andrea Panunzio:** data analysis, manuscript writing. **Alessandro Tafuri:** manuscript writing.

## Disclosure

Patents: The present investigation allows the registration of the patent‐pending invention “Pinkcare” (ITALY‐PATENT OF INVENTION Question N. 102024000014935 of 28 June 2024—Pharmaceutical or nutraceutical composition for prevention and/or treatment of uro‐gynecological disorders—Erbozeta S.p.a.). Chiara Pastorelli and Roberto Zavaglia are co‐inventors of the patent‐pending invention “Pinkcare”.

## Ethics Statement

This study was conducted in line with the principles of the Declaration of Helsinki in a clinical setting, where patients provided informed consent for the anonymous use of their data; given that the study reflects standard medical practice and all data were anonymized to protect patient privacy, we determined that formal ethical committee approval was not required.

## Conflicts of Interest

The authors declare no conflicts of interest.

## Data Availability

The data that support the findings of this study are available on request from the corresponding author. The data are not publicly available due to privacy or ethical restrictions.
